# Dialysis Duration and Glucose Exposure Amount Do Not Increase Mortality Risk in Peritoneal Dialysis Patients: A Population-Based Cohort Study From 2004 to 2012

**DOI:** 10.3389/fmed.2022.897545

**Published:** 2022-06-28

**Authors:** Pei-Yu Wu, Ming-Yen Lin, Shang-Jyh Hwang, Yi-Wen Chiu

**Affiliations:** ^1^Department of Internal Medicine, Kaohsiung Municipal Siaogang Hospital, Kaohsiung Medical University, Kaohsiung, Taiwan; ^2^Division of Nephrology, Department of Internal Medicine, Kaohsiung Medical University Hospital, Kaohsiung Medical University, Kaohsiung, Taiwan

**Keywords:** PD duration, glucose exposure, all-cause mortality, peritoneal dialysis, renal replacement therapy

## Abstract

**Background:**

Although the bio-incompatibility of glucose-based peritoneal dialysis (PD) solution is well documented, it is used worldwide. How PD duration and the amount of dialyzate glucose exposure affect survival in patients with end-stage renal disease remain inconclusive due to improper study designs in the extant literature.

**Methods:**

All incident patients with PD from 2004 to 2007 who were older than 18 years in Taiwan were included. Patients were censored when they received a transplant or at the end of 2012. Glucose exposure through PD solution was calculated by the mean glucose contained per liter when receiving PD. For those who had already shifted to hemodialysis (HD) and survived longer than 2, 3, and 4 years (the index dates), the cause-specific Cox regression model was used to make the survival comparison by PD duration and mean glucose concentration in these three cohorts, respectively. The model was adjusted by demographics, case-mix, time cohort (2004–2005 *vs.* 2006–2007), peritonitis episode (none *vs*. ≥once), and mean PD solution glucose exposure (tertile).

**Results:**

A total of 3,226 patients were included, with a mean age of 53.4 ± 15.2 years, 44.6% being male, and 34.2% having diabetes mellitus. The 1, 2, 3, and 4-year survival rates were 94, 87, 80, and 74%, while technical survival rates were 86, 70, 56, and 45%, respectively. The overall transplant events were 309 (9.6%) only. There were 389, 495, and 553 incident patients with PD shifting to HD included in 2-, 3-, and 4-year cohort, respectively. The population with moderate glucose concentration exposure had the highest mortality, and the high glucose concentration exposure had non-significant lower mortality in each cohort. In various fixed time-window cohorts, the duration of PD treatment did not increase mortality risk after adjustments. In addition, glucose exposure did not affect the mortality rate.

**Conclusion:**

For incident PD patients with PD duration no longer than 4 years, neither PD duration nor glucose exposure amount increases the long-term mortality risk.

## Introduction

Despite concerns about biocompatibility, glucose-based solutions are widely used in patients with peritoneal dialysis (PD) around the world. Although several more bio-compatible PD solutions are now commercially available, none of them have been proven beneficial in terms of hard outcomes, such as mortality (1–4). Given the high cost of new solutions, the glucose-based solution remains the first choice of PD prescription worldwide. Long-term exposure to the traditional glucose-based solution, as expected, injures the peritoneal membrane substantially because of its acidic pH, high lactate, osmolality, glucose, and glucose degradation products (5–7). The whole mechanism of harm from PD solutions remains complicated. All aforementioned stimulants trigger angiogenesis and fibrogenesis in the peritoneum, damage membrane solute transport, provoke residual renal function loss, and further cause PD technical failure, as well as the rare but fatal encapsulated peritoneum sclerosis (EPS) (8–14). Since the injuries above to the peritoneum are irreversible and in progression even after PD cessation (15, 16), a major concern exists that the amount and duration of glucose exposure to the peritoneum might affect clinical outcomes, and whether or not there should be a planned stoppage of PD before reaching technical failure (17).

However, few studies have investigated how PD glucose exposure affects clinical outcomes, such as technical survival (12, 18), all-cause mortality (12, 19), and cardiovascular (CV) mortality (19), and even then the results were inconsistent. The reason for this might be that PD glucose exposure is a time-dependent variable. When testing the effect of the PD duration and total glucose exposure in the whole PD course on the clinical outcomes, we need to count every dialyzate glucose exposure in all PD duration. Instead of using an initial PD prescription or time-average concentration to represent baseline glucose exposure, as other studies did, we created three cohorts by different fixed time windows. In each time window, all participants had ended the PD therapy and shifted to HD, and thus all PD duration and total glucose exposure were measured (12, 18–20). Furthermore, irrespective of whether the incident or enrollment date is set as the index day, PD exposure duration is measured imprecisely in these studies because PD treatment is continuously given during the observation period (12, 18–22). Therefore, we conducted a study using the fixed time-window to avoid those time biases. Specifically, we only included those patients with PD who had shifted to hemodialysis (HD) on the index date to determine whether PD duration or amount of dialyzate glucose exposure would be associated with the risk of mortality in incident patients with PD.

## Materials and Methods

### Design and Data Sources

We conducted a retrospective, population-based cohort study using the National Health Insurance Research Database (NHIRD), which is the claim database of the single-payer insurance system in Taiwan, with a coverage rate of more than 99%. The NHIRD comprises highly accurate information about diagnostic codes, drug prescriptions, and medical procedures, but no laboratory results, from contracted healthcare institutes. It has been well applied for generating real-world population-based evidence to test clinical and epidemiological research hypotheses (23). The Registry of Catastrophic Illness is a national registry system under the Taiwan National Health Insurance for patients with catastrophic illnesses, such as end-stage renal disease requiring renal replacement therapy, to waive co-payments or deductions for dialysis-related fees to reduce barriers to medical care.

### Study Participants

From February 2004 to December 2007, we included all incident patients with PD from the NHIRD according to the following criteria. First, one should be issued the Registry of Catastrophic Illness and identified by the International Classification of Disease, Ninth Revision, Clinical Modification (ICD-9-CM) codes 585, 403.01, 403.11, 403.91, 404.02, 404.03, 404.12, 404.13, 404.92, or 404.93. Then, subjects should have maintenance dialysis longer than 90 days, and have a PD prescription within 90 days after the first dialysis. Patients who did not have a birth record, were less than 18-years-old, and had a renal transplant history were excluded. A total of 3,226 patients formed the original study cohort. All study subjects were followed until death or the end of observation on 31 December 2012. This study was approved by the Institutional Review Board of the Kaohsiung Medical University Hospital, Kaohsiung Medical University, Taiwan (KMUHIRB-E(I)-20190137). All research procedures followed the directives of the Declaration of Helsinki. Since all identifiable privacy information was encrypted with unique and anonymous identifiers, the review board waived the requirement for informed written consent.

### Definitions of Different Time Cohorts, Peritoneal Dialysis Glucose Exposure, and Death

**FIGURE 1:**
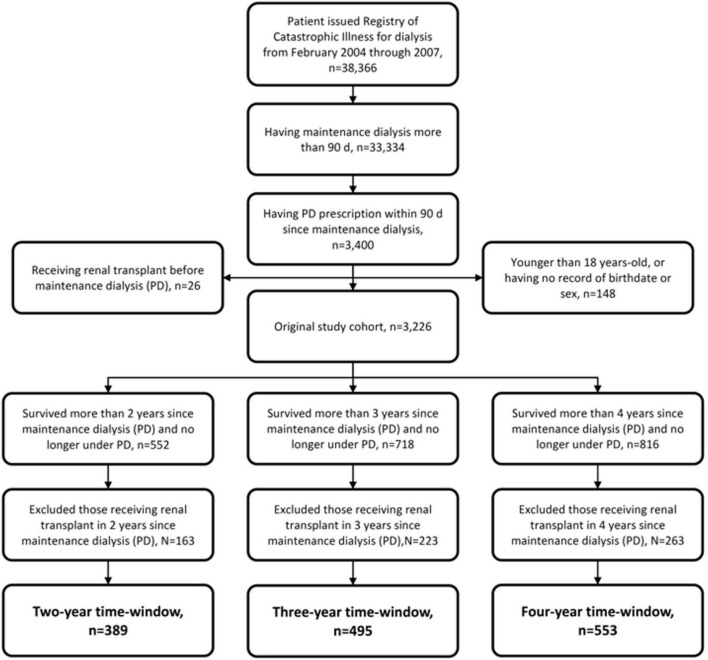
Flowchart of patient enrollment in different fixed time-window cohorts.

To avoid time-window bias resultant from PD duration, we created three study time cohorts to evaluate the effects of PD duration and PD glucose on long-term mortality. We defined 2-, 3-, and 4-year cohorts as including incident patients with PD who had already shifted to HD and survived longer than 2, 3, and 4 years since the initiation of PD, respectively. The detailed patient selection process is shown in Figure 1. For further survival comparison, PD exposure duration within different fixed time-windows was categorized as: <1 and 1–2 years in the 2-year cohort; <1, 1–2, and 2–3 years in the 3-year cohort; and <1, 1–2, 2–3, and 3–4 years in the 4-year cohort.

We determined the exposure of glucose in PD dialyzate using mean glucose concentration (mg/dl), defined as total accumulated glucose in PD dialyzate divided by total accumulated PD solution volume during PD treatment. The glucose amount of icodextrin was calculated as zero. We further classified mean glucose concentration into tertiles in each time cohort for further analysis. All-cause mortality was defined as either the presence of a death date or no other renal replacement therapy treatment claim that could be traced in the dataset.

### Covariates

Patient demographic characteristics of age, sex, socioeconomic status [dependent, <20,000, and ≥20,000 New Taiwan dollars (approximately 667 United States dollars)], and resident urbanization (rural and urban) were collected from the NHIRD. We also identified patients’ major comorbidities, such as diabetes mellitus (DM) (ICD-9-CM code: 250), hypertension (ICD-9-CM codes: 401–405), myocardial infarction (ICD-9-CM code: 410), heart failure (ICD-9-CM code: 428), cerebrovascular disease (ICD-9-CM codes: 433, 434, and 436), gout (ICD-9-CM code: 274), and peripheral vascular disease (ICD-9-CM codes: 250.7, 443.9, 442.2, 785.4, 443.00, and 443.81). The presence of comorbidity was defined as that the defined diagnostic code appeared ≥2 times in outpatient claims or ≥1 time in admission claims within 1 year prior to PD initiation. Disease severity was displayed using the Charlson comorbidity index, which was calculated based on the formula from previous literature (24). In addition, PD-related peritonitis was considered to be an important confounder, and identified by the combination of ICD-9-CM code (996.68) and the prescription of intravenous-form antibiotics.

### Statistical Analyses

Data were presented as mean ± standard deviation (SD) or median with interquartile range (IQR) for continuous variables and percentage for categorical variables as appropriate. The follow-up time for mortality analyses in each cohort began at the end of the fixed time-window until death or the end of observation on 31 December 2012, which ever occurred first. We excluded incident patients with PD receiving renal transplants in the fixed time window, and censored them on the date of surgery when their renal transplants occurred during the observation period. Cumulative risks of the mortality were estimated using the Kaplan–Meier survival analysis, and the Wilcoxon test examined differences in risks of those exposed to different PD durations and concentrations of glucose-based PD dialyzate. By taking the renal transplant event as a competitive risk, the cause-specific Cox regression model adjusting for the covariates was applied to explore the associations of PD duration and concentration of glucose-based PD dialyzate with the risk of all-cause mortality. Interactions of PD duration and glucose concentration with all-cause mortality were also inspected in the models, and they were introduced into the model once the interaction above showed significance. Among the covariates included in the cause-specific Cox model were age, sex, socioeconomic status, resident urbanization, comorbidities, Charlson comorbidity index, PD duration, total glucose exposure through PD dialyzate, and PD-related peritonitis. We further adjusted our model with vascular access type and HD center size as a sensitivity test. To test the time effect on mortality after the fixed time-window, we further stratified the observation period of each cohort based on its duration, which was: <1, 1–2, 2–3, 3–4, and >4 years for the 2-year cohort; <1, 1–2, 2–3, and >3 years for the 3-year cohort; and <1, 1–2, and >2 years for the 4-year cohort. The adjusted hazard ratio (*AHR*) was calculated for every time frame above in each cohort. We further divided each cohort by DM status and reran the analysis. All statistical operations were performed using SAS (version 9.4, SAS Institute, Cary, NC, United States). A *p*-value < 0.05 was defined as statistically significant.

## Results

A total of 3,226 incident patients with PD were enrolled from 2004 to 2007, with the baseline characteristics presented in Table 1. The median PD duration was approximately 4 years. A total of 1,331 (41.3%) patients had suffered from PD-related peritonitis at least once in the fixed time-window periods, and the total transplant events were 309 (9.6%). The survival rates of 1, 2, 3, and 4 years were 94, 87, 80, and 70%, respectively; while the technical survival rates of 1, 2, 3, and 4 years were 86, 70, 56, and 45%, respectively. By the definition of 2-, 3-, and 4-year cohort in this study, there were 389, 495, and 553 incident patients with PD included in each cohort, respectively (Figure 1); these were the results of balance among patient survival, PD technical survival, and receiving a renal transplant or not. Compared with the overall study population, any variable relevant to those three factors mentioned above (patient survival, PD technical survival, and receiving a renal transplant or not) would probably significantly differ among the three cohorts. For instance, patients from the 2- to 4-year cohort were: younger; more male; less having DM, myocardial infarction, and congestive heart failure; and lower comorbidity burden. As anticipated, median PD duration increased as the cohort went from 2- to 4-year.

**TABLE 1:** Patient characteristics in overall and different fixed time-window cohorts.

Characteristics		Fixed time-window cohorts		
	Overall	Two-year^a^	Three-year^b^	Four-year^c^
Number	3,226	389	495	553
Age^#,$,&^ (year)	53.4(±15.2)	55.3(±14.6)	52.8(±15)	51.9(±14.5)
Male sex^&^ (%)	1439(44.6%)	172(44.2%)	236(47.7%)	272(49.2%)
**Socioeconomic status**				
Dependent (%)	391(12.1%)	53(13.6%)	66(13.3%)	72(13%)
<20,000 NTD (%)	1691(52.4%)	213(54.8%)	266(53.7%)	290(52.4%)
≥20,000 NTD (%)	1144(35.5%)	123(31.6%)	163(32.9%)	191(34.5%)
**Urbanization**				
Rural (%)	663(20.6%)	90(23.1%)	110(22.2%)	120(21.7%)
Urban (%)	2563(79.4%)	299(76.9%)	385(77.8%)	433(78.3%)
**Comorbidity**				
Diabetes mellitus^#,$^ (%)	1103(34.2%)	184(47.3%)	212(42.8%)	210(38%)
Hypertension (%)	2586(80.2%)	314(80.7%)	405(81.8%)	452(81.7%)
Myocardial infarction (%)	53(1.6%)	7(1.8%)	8(1.6%)	6(1.1%)
Congestive heart failure^#^ (%)	429(13.3%)	70(18.0%)	78(15.8%)	79(14.3%)
Stroke (%)	158(4.9%)	23(5.9%)	26(5.3%)	23(4.2%)
Gout (%)	596(18.5%)	81(20.8%)	100(20.2%)	113(20.4%)
Peripheral vascular disease (%)	104(3.2%)	18(4.6%)	20(4%)	23(4.2%)
Charlson index^#,$,&^	3(2,5)	4(2,6)	3(2,5)	3(2,5)
**Charlson index, by score^#,$^**				
2	92(2.9%)	8(2.1%)	12(2.4%)	12(2.2%)
3–4	1032(32%)	91(23.4%)	127(25.7%)	163(29.5%)
5–6	1159(35.9%)	141(36.2%)	180(36.4%)	207(37.4%)
≥7	943(29.2%)	149(38.3%)	176(35.6%)	171(30.9%)
PD duration^#,$,&^ (day)	1383(599.5,2122)	362(153,575)	548(262,821)	690(329,1034)
PD-related peritonitis^$,&^ (%)	1331(41.3%)	174(44.7%)	245(49.5%)	279(50.5%)
Peritonitis rate (per person-year) (95%CI)	0.179(0.155,0.207)	0.119(0.099,0.142)	0.129(0.109,0.153)	0.123(0.103,0.147)

*NTD, new Taiwan dollar; PD, peritoneal dialysis; CI, confidence interval.*

*^a^PD duration less than 2 years and survival of more than 2 years, i.e., a 2-year cohort.*

*^b^PD duration less than 3 years and survival of more than 3 years, i.e., a 3-year cohort.*

*^c^PD duration less than 4 years and survival of more than 4 years, i.e., a 4-year cohort.*

*Data are represented as mean ± standard deviation (SD) or median (interquartile range) for continuous variables and count (proportion) for categorical variables. An independent t-test or Mann–Whitney U-test and χ^2^ are applied for comparing the differences of continuous and categorical variables, respectively, among the fixed time-window cohorts and overall cohort.*

*^#^p < 0.05 compared to the overall cohort.*

*^$^p < 0.05 compared to the overall cohort.*

*^&^p < 0.05 compared to the overall cohort.*

**FIGURE 2:**
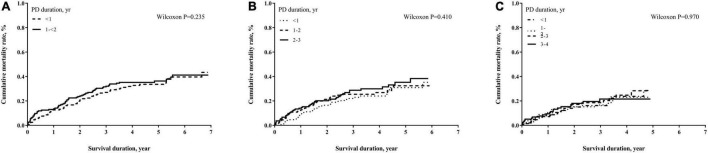
Cumulative mortality proportion by peritoneal dialysis (PD) duration. **(A)** Cumulative mortality proportion in the 2-year cohort; **(B)** cumulative mortality proportion in the 3-year cohort; **(C)** cumulative mortality proportion in the 4-year cohort. The Kaplan–Meier approach was used to estimate the mortality proportion, and the differences of mortality proportion were examined by the Wilcoxon test. A *p*-value < 0.05 was significant.

**FIGURE 3:**
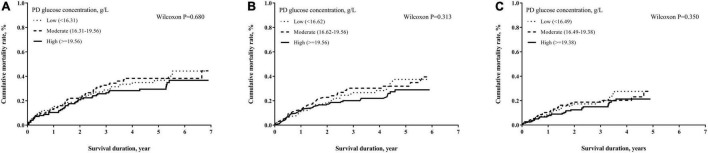
Cumulative mortality proportion by mean glucose concentration of PD dialyzate. **(A)** Cumulative mortality proportion in the 2-year cohort; **(B)** cumulative mortality proportion in the 3-year cohort; **(C)** cumulative mortality proportion in the 4-year cohort. The Kaplan–Meier approach was used to estimate the mortality proportions with the differences of mortality proportion examined by the Wilcoxon test. A *p*-value < 0.05 was significant.

The numbers of deaths, observation period, and mortality rate in different time cohorts by PD duration and exposed glucose concentration are summarized in Table 2. The number of deaths and mortality rate decreased from 2- to 4-year cohort. In addition, a non-significant trend of a higher mortality rate in subjects with longer PD duration in each time cohort was observed. Mean glucose concentrations in each tertile were not significantly different among these three cohorts. However, the population with moderate glucose concentration exposure had the highest mortality, and the high glucose concentration exposure had non-significant lower mortality in each time cohort. When we used the Kaplan–Meier approach to estimate the cumulative mortality in these three cohorts, neither PD duration nor PD glucose concentration significantly impacted mortality outcome (Figures 2, 3). However, for patients with DM in all three cohorts, the population with a longer PD duration had a higher mortality but not the non-DM population (Supplementary Tables 2-1, 2-2).

**TABLE 2:** Death event number, observation period, and mortality concerning peritoneal dialysis exposure by different time-window cohort.

Parameters	Patient number	Number of deaths	Observation period*, patient-years	Mortality rate (95% CI), per 1,000 patient-years	*p*-value^&^

2-year cohort (*n* = 389)					
**P.D. duration (year)**					
<1	199	68	729.1	93.3(73.5,118.3)	Reference
1–2	190	69	649.0	106.3(84.0,134.6)	0.44
**Mean PD glucose concentration^#^ (mg/dL)**					
Low (<1.63)	128	45	433.9	103.7(77.4,138.9)	Reference
Moderate (1.63–1.96)	132	50	454.8	109.9(83.3,145.1)	0.78
High (>1.96)	129	42	489.5	85.8(63.4,116.1)	0.37

**3-year cohort (*n* = 495)**					

**PD duration (year)**					
<1	172	43	544.0	79.0(58.6,106.6)	Reference
1–<2	157	43	485.0	88.7(65.8,119.5)	0.59
2–3	166	49	487.1	100.6(76.0,133.1)	0.25
**Mean PD glucose concentration^#^ (mg/dL)**					
Low (<1.66)	163	45	475.9	94.6(70.6,126.6)	Reference
Moderate (1.66–1.96)	168	51	495.8	102.9(78.2,135.3)	0.68
High (>1.96)	164	39	544.4	71.6(52.3,98.1)	0.20

**4-year cohort (*n* = 553)**					

**PD duration (year)**					
<1.0	156	28	377.9	74.1(51.2,107.3)	Reference
1–<2	136	24	340.4	70.5(47.3,105.2)	0.86
2–<3	140	26	337.2	77.1(52.5,113.2)	0.88
3–4	121	21	272.3	77.1(50.3,118.3)	0.89
**Mean PD glucose concentration^#^ (mg/dL)**					
Low (<1.65)	182	34	414.0	82.1(58.7,114.9)	Reference
Moderate (1.65–1.94)	188	36	431.4	83.5(60.2,115.7)	0.95
High (>1.94)	183	29	482.4	60.1(41.8,86.5)	0.22

*CI, confidence interval; PD, peritoneal dialysis.*

*Calculated from the end of fixed time-windows, 2-, 3-, and 4-year, respectively, to the time of death or 31 December 2012.

*^&^The different mortality rates by PD duration and glucose concentration in each time cohort were tested by the Poisson regression model.*

*^#^Counted by using total accumulated glucose in PD dialyzate divided by total accumulated PD solution volumes during PD treatment.*

In Table 3, we used a cause-specific Cox model analysis to test the effect of PD duration and PD glucose concentration on mortality in all three cohorts. After adjusting for all variables listed in Table 1, it was found that the *AHR* increased as PD duration increased. Surprisingly, the high PD glucose concentration group had a lower *AHR* than the moderate one when using the low one as a reference in two of the three cohorts. However, none of them was statistically significant, indicating that we cannot find any increased mortality risk by either longer PD duration or higher PD glucose concentration in our 2-, 3-, or 4-year cohorts. The results were similar even after further adjusting for vascular access type and HD center size (Supplementary Table 4). Furthermore, there is no interaction of PD duration and glucose concentration in the models when testing mortality after the fixed time-windows. Other risk factors associated with all-cause mortality included age, DM, peripheral vascular disease, number of peritonitis, and Charlson comorbidity index. Among them, only age was consistently significant in all three cohorts (Supplementary Table 1). Given that our data do not meet the proportional hazard assumption in the current Cox model, we further stratified the observation period based on length to determine mortality hazard in different time frames. The results are presented in Table 4. The hazard ratios (*HR*s) of mortality by observation duration were not statistically significant among different mean glucose concentration exposure except for one, ≥4 year in the 2-year cohort. Similarly, the 3- and 4-year cohorts had only one significant result among different PD durations but no dose-response. Similar to our prior results, neither PD duration nor mean glucose exposure affects most of the mortality in DM and non-DM cohorts after adjustment (Supplementary Tables 3-1, 3-2), with the exception of the PD duration in the 4-year cohort of patients with DM (*HR*: 3.50, 95% *CI*: 1.58–7.75).

**TABLE 3:** Risk of mortality concerning PD exposure by different time-window cohorts by the cause-specific Cox regression model^#^.

Parameters	Crude hazard ratio (95% CI)	Adjusted hazard ratio (95% CI)	*p-*value

2-year cohort (*n* = 389)			
**PD duration (year)**			0.62
<1	Reference	Reference	
1–2	1.14 (0.82, 1.60)	0.91 (0.62, 1.33)	0.62
**Mean PD glucose concentration (mg/dL)**			0.71
Low (<1.63)	Reference	Reference	
Moderate (1.63–1.96)	1.05 (0.70, 1.57)	0.84 (0.53, 1.33)	0.45
High (>1.96)	0.86 (0.57, 1.31)	0.84 (0.52, 1.37)	0.49

**3-year cohort (*n* = 495)**			

**PD duration (year)**			0.41
<1	Reference	Reference	
1–2	1.13 (0.74, 1.73)	0.98 (0.63, 1.53)	0.94
2–3	1.26 (0.84, 1.90)	1.29 (0.82, 2.02)	0.27
**Mean PD glucose concentration (mg/dL)**			0.67
Low (<1.66)	Reference	Reference	
Moderate (1.66–1.96)	1.10 (0.74, 1.65)	0.84 (0.54, 1.31)	0.44
High (>1.96)	0.80 (0.52, 1.22)	0.83 (0.52, 1.33)	0.43

**4-year cohort (*n* = 553)**			

**PD duration (year)**			0.52
<1	Reference	Reference	
1–2	0.96 (0.56, 1.66)	0.87 (0.48, 1.55)	0.63
2–3	1.03 (0.60, 1.76)	1.35 (0.76, 2.38)	0.30
3–4	1.03 (0.58, 1.81)	1.09 (0.57, 2.09)	0.80
**Mean PD glucose concentration (mg/dL)**			0.75
Low (<1.65)	Reference	Reference	
Moderate (1.65–1.94)	1.02 (0.64, 1.63)	0.92 (0.55, 1.55)	0.76
High (>1.94)	0.76 (0.46, 1.25)	0.81 (0.46, 1.41)	0.45

*^#^Renal transplant as a competing event.*

*CI, confidence interval; PD, peritoneal dialysis.*

*Model adjusted for age, sex, socioeconomic status, urbanization, comorbidity (diabetes mellitus, hypertension, myocardial infarction, congestive heart failure, stroke, gout, and peripheral vascular disease), Charlson score, and PD-related peritonitis (categorized by none, one time, and more than one time).*

**TABLE 4:** Risk of mortality concerning PD exposure by follow-up duration in three different time-window cohorts by the cause-specific Cox regression model^#^.

Parameters	Follow-up duration (year)									
	Adjusted HR (95% CI)	*p-*value	Adjusted HR (95% CI)	*p*-value	Adjusted HR (95% CI)	*p*-value	Adjusted HR (95% CI)	*p*-value	Adjusted HR (95% CI)	*p*-value
2-year cohort (*n* = 389)	<1		1–2		2–3		3–4		≥4	
PD duration (year)		0.44		0.30		0.47		0.12		0.09
<1	Reference		Reference		Reference		Reference		Reference	
1–2	1.26 (0.71–2.25)	0.44	1.47 (0.71–3.02)	0.30	1.36 (0.60–3.11)	0.47	0.27 (0.05–1.43)	0.12	0.14 (0.02–1.31)	0.09
Mean PD glucose concentration (mg/dL)		0.65			0.77		0.44		0.20	0.07
Low (<1.63)	Reference		Reference		Reference		Reference		Reference	
Moderate (1.63–1.96)	0.72 (0.35–1.46)	0.36	1.13 (0.43–2.94)	0.81	1.39 (0.51–3.78)	0.52	0.49 (0.10–2.39)	0.38	0.03 (0.001–0.67)	0.03
High (>1.96)	0.91 (0.44–1.89)	0.80	1.39 (0.54–3.62)	0.50	0.71 (0.22–2.32)	0.57	0.13 (0.01–1.23)	0.08	0.24 (0.04–1.59)	0.14
3-year cohort (*n* = 495)	<1		1–2		2–3		≥3			
PD duration (year)		0.31		0.71		0.01		0.47	–	
<1	Reference		Reference		Reference		Reference			
1–2	1.34 (0.67, 2.69)	0.41	1.40 (0.61, 3.21)	0.42	0.49 (0.14, 1.66)	0.25	0.41 (0.10–1.70)	0.22		
2–3	1.71 (0.86, 3.38)	0.12	1.33 (0.54, 3.26)	0.54	2.84 (1.01, 7.96)	0.05	0.65 (0.14–3.02)	0.59		
Mean PD glucose concentration (mg/dL)		0.78			0.14		0.25	0.21		
Low (<1.66)	Reference		Reference		Reference		Reference		–	
Moderate (1.66–1.96)	0.79 (0.40, 1.55)	0.50	1.73 (0.77, 3.89)	0.19	1.01 (0.38, 2.71)	0.99	0.23 (0.04–1.18)	0.08		
High (>1.96)	0.93 (0.46, 1.87)	0.83	0.75 (0.29, 1.99)	0.57	0.36 (0.10, 1.31)	0.12	0.55 (0.15–1.98)	0.36		
4-year cohort (*n* = 553)	<1		1–2		≥2					
PD duration (year)		0.70		0.03		0.47	–			
<1	Reference		Reference		Reference					
1–2	1.35 (0.61, 2.99)	0.46	0.48 (0.15, 1.57)	0.23	0.52 (0.13–2.08)	0.35				
2–3	1.21 (0.51, 2.89)	0.67	2.13 (0.81, 5.65)	0.13	0.98 (0.24–4.06)	0.98				
3–4	1.67 (0.71, 3.93)	0.24	2.27 (0.79, 6.55)	0.13	0.25 (0.04–1.77)	0.17	–			
Mean PD glucose concentration (mg/dL)		0.69		0.44		0.12				
Low (<1.65)	Reference		Reference		Reference					
Moderate (1.65–1.94)	1.13 (0.56, 2.31)	0.73	0.98 (0.41, 2.31)	0.96	0.18 (0.04, 0.92)	0.04				
High (>1.94)	0.82 (0.36, 1.83)	0.62	0.54 (0.20, 1.52)	0.25	0.64 (0.20, 2.04)	0.45				

*^#^Renal transplant as a competing event.*

*CI, confidence interval; PD, peritoneal dialysis.*

*Model adjusted for age, sex, socioeconomic status, urbanization, comorbidity (diabetes mellitus, hypertension, myocardial infarction, congestive heart failure, stroke, gout, and peripheral vascular disease), Charlson score, and PD-related peritonitis (categorized by none, one time, and more than one time).*

## Discussion

To the best of the authors’ knowledge, our study is the first to investigate the impact of PD duration and PD glucose exposure on mortality risk in incident patients with PD who had already shifted to maintenance HD prior to the study index date. This study avoids the time-dependent bias and calculates total glucose exposure during PD treatment, which are ignored in almost all extant literature. We found that neither longer PD duration nor higher glucose exposure amount increases the risk for all-cause mortality in incident patients with PD having PD treatment less than 4 years and already shifted to HD treatment. The results were the same during reanalysis after stratifying the observation time. As expected, older age increased the risk for all-cause mortality in our study cohorts (12, 19).

Although the association of PD duration and mortality has long been investigated, no consistent conclusion has been reached (21, 22, 25). The main reason for this is that PD duration has seldom been correctly defined. When using time-to-event analysis, time bias will occur if the study design allows PD treatment to exist after the index day, i.e., during the observation period. Irrespective of including incident or prevalent patients with PD, the authors tend to make three kinds of errors in those studies. The first error is to ignore the PD treatment during the observation period, regardless of the treatment length (12, 18–22, 25, 26). The second is to censor the PD participants when shifting to HD, and such study design excludes those mortalities occurring after the change of renal replacement therapy modality, which is highly common (20, 21, 25).

This also causes time-to-event analysis to show that the longer PD duration was associated with death because the incident patients with PD tend to shift to HD or have renal transplants rather than die on PD. The third, and most important, error is that those studies cannot distinguish the effect on mortality of PD treatment from uremic exposure period (18–22, 25). The longer PD duration before enrollment also means more extended exposure to uremia, which is associated with a higher mortality risk and needs further adjustment. However, to the best of the authors’ knowledge, it is almost impossible to distinguish the effect of PD duration from the duration of uremic status exposure on all-cause mortality in the aforementioned design. In other words, once the time-to-event study sets the index day before the cessation of PD treatment, regardless of whether incident or prevalent patients are included, such a design will confound the effect of PD treatment and uremic exposure duration on mortality. Indeed, this makes one less likely to discern the pure PD treatment effect on mortality, which constitutes a crucial issue for both patients with PD and care teams. To overcome all of the disadvantages in the above-mentioned methodology, we used a fixed time-window to observe the long-term effect of PD exposure on survival in patients who already shifted to HD. In our study design, uremic exposure is also well-controlled, which assists us to precisely define the PD treatment effect on all-cause mortality. In PD exposure of less than 4 years, we cannot confirm that all-cause mortality increases after stopping PD.

Given the correct measurement of PD duration in our cohort, we can further test the effect of total PD glucose exposure on mortality. In extant literature, including incident patients with PD and calculating PD glucose exposure by using glucose concentration in the beginning or short-term observation period, may not represent total glucose load during the follow-up period (12, 19, 20, 26). This kind of study design may not provide a precise evaluation of the total PD glucose effect on the patients’ outcomes. At best, they only used baseline PD glucose concentration to predict mortality, and even then they reported inconsistent results. By including only subjects who had already shifted to HD, our study could precisely calculate total PD duration and PD dialyzate glucose exposure. Such a study design assists us to avoid the changes of both factors during the observation periods influencing the outcome. Considering the deeply interactive effect of PD duration and dialyzate glucose concentration on clinical outcomes, one cannot evaluate the effect of glucose exposure without correctly measuring PD duration. Based on controlling PD duration, our results cannot demonstrate that higher PD glucose concentration can increase mortality risk.

Peritoneal fibrosis is inevitable after exposure to bio-incompatible PD dialyzate; whereas, peritoneal technical failure usually occurs afterward. It remains unclear how the damage of peritoneum after exposure to PD dialyzate affects long-term outcomes. Both uremia and PD dialyzate exposure induce peritoneal membrane injury, and longer PD vintage is undoubtedly linked to the progressive morphological and histological change of the peritoneal membrane (27–31). In terms of the clinical outcome, however, our results did not support more prolonged PD treatment, or that higher glucose exposure increased mortality risk. The causes of mortality in PD are both numerous and complex. Indeed, age, CV disease, DM, PD peritonitis episodes, higher body mass index, higher alkaline phosphatase, inflammation, and anemia are all associated with PD mortality (32). The small effect size of PD duration and PD glucose load on mortality may be one of the reasons that the result was not significant in our study. However, one study reports a similar finding that higher blood sugar in non-DM PD patients was not associated with higher mortality (33). It is also worth noting that, prior to using our findings to make clinical decisions, one should be cognizant that not increasing mortality risk is not equivalent to less harm.

The strengths of this study are its design, and we have created three specific time cohorts of different fixed time-windows to avoid the time bias. We classified PD duration and glucose exposure concentration in the selected time-window and used them as the baseline dependent variables to avoid any time bias when performing the time-to-event analysis. This study also possesses certain limitations. First, this is a retrospective study from the NHIRD from 2004 to 2012 in Taiwan. There is no information on laboratory data, such as fasting glucose and glycated hemoglobin levels, and technical failure causes. Even the baseline patient characteristics, PD penetration, PD prescription, and PD policy did not change much after 2012, one should be careful when generalizing the results. Second, we calculated glucose concentration according to the PD solution prescription, but we could not ensure patients’ adherence, which could lead to overestimated glucose concentration exposure. Third, we excluded those who died under PD treatment, and this made the result unable to be applied to all patients with PD. The clinical application of our findings concerns determination of whether further PD treatment will increase mortality risk, and thus only those who survive in PD need to be considered. Fourth, we did not evaluate the effect of non-glucose peritoneal dialyzate solution, such as icodextrin. There is no sufficient evidence, however, to prove that these dialyzate solutions could improve the outcome. Fifth, based on our study design, some mortalities in the transition from PD to HD were not included for analysis since these events occurred in the fixed time windows. Finally, we have omitted HD-related covariates, such as vascular access type, HD compliance, and KT/V, for adjusting our COX model to predict all-cause mortality. However, we had put vascular access type and HD center size into the model for adjustment as a sensitive test and found similar results (Supplementary Table 4). A large prospective study is necessary to transcend the above limitations.

In conclusion, we selected incident patients with PD who had already shifted to HD to estimate glucose exposure precisely and included patients who survived longer than the time window to exclude time bias. We proved that neither PD duration nor glucose exposure increased the risk for all-cause mortality in the group of PD duration less than 4 years. The 4-year technical survival rate was 45% in the NHIRD, and this result may be interpreted as demonstrating the relative safety of glucose dialyzate use in more than half of patients with PD in Taiwan. Surprisingly, in the 4-year cohort, the DM subgroup had their PD duration significantly associated with mortality. It could be that higher mortality occurred in the transition from PD to HD was more included in the longer PD duration group based on our study design. We need a larger and longer dataset to confirm whether patients with DM have higher mortality if they stay in PD longer.

## Data Availability Statement

The data analyzed in this study is subject to the following licenses/restrictions: The data underlying this study is from National Health Insurance Research Database (NHIRD). Due to restrictions placed on the data by Taiwan National Health Insurance, the minimal data set cannot be made publicly available. Requests to access these datasets should be directed to Pei-Yu Wu, wpuw17@gmail.com.

## Ethics Statement

The studies involving human participants were reviewed and approved by the Institutional Review Board of Kaohsiung Medical University Hospital, Kaohsiung Medical University, Taiwan (KMUHIRB-E(I)-20190137). Written informed consent for participation was not required for this study in accordance with the national legislation and the institutional requirements.

## Author Contributions

P-YW, M-YL, S-JH, and Y-WC contributed to conceptualization. M-YL contributed to methodology. S-JH contributed to validation. P-YW contributed to investigation, writing—original draft preparation, and project administration. Y-WC contributed to writing—review and editing. Y-WC and S-JH contributed to supervision. P-YW and Y-WC contributed to funding acquisition. All authors have read and agreed to the published version of the manuscript.

## Conflict of Interest

The authors declare that the research was conducted in the absence of any commercial or financial relationships that could be construed as a potential conflict of interest.

## Publisher’s Note

All claims expressed in this article are solely those of the authors and do not necessarily represent those of their affiliated organizations, or those of the publisher, the editors and the reviewers. Any product that may be evaluated in this article, or claim that may be made by its manufacturer, is not guaranteed or endorsed by the publisher.

## References

[1] Kim S Oh J Kim S Chung W Ahn C Kim SG Benefits of biocompatible Pd fluid for preservation of residual renal function in incident Capd patients: a 1-year study. *Nephrol Dial Transplant.* (2009) 24:2899–908. 10.1093/ndt/gfp054 19258384

[2] Lai KN Lam MF Leung JC Chan LY Lam CW Chan IH A study of the clinical and biochemical profile of peritoneal dialysis fluid low in glucose degradation products. *Perit Dial Int.* (2012) 32:280–91. 10.3747/pdi.2010.00176 22045098 PMC3525436

[3] Cho Y Johnson DW Badve S Craig JC Strippoli GF Wiggins KJ. Impact of icodextrin on clinical outcomes in peritoneal dialysis: a systematic review of randomized controlled trials. *Nephrol Dial Transplant.* (2013) 28:1899–907. 10.1093/ndt/gft050 23493329

[4] Szeto CC Kwan BC Chow KM Cheng PM Kwong VW Choy AS The effect of neutral peritoneal dialysis solution with low glucose-degradation-product on the fluid status and body composition–a randomized control trial. *PLoS One.* (2015) 10:e0141425. 10.1371/journal.pone.0141425 26510186 PMC4625015

[5] Ito T Yorioka N Yamamoto M Kataoka K Yamakido M. Effect of glucose on intercellular junctions of cultured human peritoneal mesothelial cells. *J Am Soc Nephrol.* (2000) 11:1969–79. 11053471 10.1681/ASN.V11111969

[6] Cendoroglo M Sundaram S Jaber BL Pereira BJ. Effect of glucose concentration, osmolality, and sterilization process of peritoneal dialysis fluids on cytokine production by peripheral blood mononuclear cells and polymorphonuclear cell functions in vitro. *Am J Kidney Dis.* (1998) 31:273–82. 10.1053/ajkd.1998.v31.pm9469498 9469498

[7] Witowski J Korybalska K Wisniewska J Breborowicz A Gahl GM Frei U Effect of glucose degradation products on human peritoneal mesothelial cell function. *J Am Soc Nephrol.* (2000) 11:729–39. 10.1681/ASN.V114729 10752532

[8] Boulanger E Wautier MP Wautier JL Boval B Panis Y Wernert N Ages bind to mesothelial cells via rage and stimulate Vcam-1 expression. *Kidney Int.* (2002) 61:148–56. 10.1046/j.1523-1755.2002.00115.x 11786095

[9] Nakamura S Tachikawa T Tobita K Miyazaki S Sakai S Morita T Role of advanced glycation end products and growth factors in peritoneal dysfunction in capd patients. *Am J Kidney Dis.* (2003) 41(3 Suppl 1):S61–7. 10.1053/ajkd.2003.50087 12612955

[10] Davies SJ Phillips L Naish PF Russell GI. Peritoneal glucose exposure and changes in membrane solute transport with time on peritoneal dialysis. *J Am Soc Nephrol.* (2001) 12:1046–51. 10.1681/ASN.V1251046 11316864

[11] Szeto CC Kwan BC Chow KM Chung S Yu V Cheng PM Predictors of residual renal function decline in patients undergoing continuous ambulatory peritoneal dialysis. *Perit Dial Int.* (2015) 35:180–8. 10.3747/pdi.2013.00075 24497594 PMC4406313

[12] Wu HY Hung KY Huang TM Hu FC Peng YS Huang JW Safety issues of long-term glucose load in patients on peritoneal dialysis–a 7-year cohort study. *PLoS One.* (2012) 7:e30337. 10.1371/journal.pone.0030337 22303440 PMC3264614

[13] Nakayama M Kawaguchi Y Yamada K Hasegawa T Takazoe K Katoh N Immunohistochemical detection of advanced glycosylation end-products in the peritoneum and its possible pathophysiological role in Capd. *Kidney Int.* (1997) 51:182–6. 10.1038/ki.1997.22 8995732

[14] Nakamoto H Hamada C Shimaoka T Sekiguchi Y Io H Kaneko K Accumulation of advanced glycation end products and beta 2-microglobulin in fibrotic thickening of the peritoneum in long-term peritoneal dialysis patients. *J Artif Organs.* (2014) 17:60–8. 10.1007/s10047-013-0741-1 24337623

[15] Brown MC Simpson K Kerssens JJ Mactier RA Scottish Renal R. Encapsulating peritoneal sclerosis in the new millennium: a national cohort study. *Clin J Am Soc Nephrol.* (2009) 4:1222–9. 10.2215/CJN.01260209 19541815 PMC2709523

[16] Kawanishi H Kawaguchi Y Fukui H Hara S Imada A Kubo H Encapsulating peritoneal sclerosis in japan: a prospective, controlled, multicenter study. *Am J Kidney Dis.* (2004) 44:729–37. 15384025

[17] Brown EA Bargman J van Biesen W Chang MY Finkelstein FO Hurst H Length of time on peritoneal dialysis and encapsulating peritoneal sclerosis - position paper for ispd: 2017 update. *Perit Dial Int.* (2017) 37:362–74. 10.3747/pdi.2017.00018 28676507

[18] Wu HY Hung KY Huang JW Chen YM Tsai TJ Wu KD. Initial glucose load predicts technique survival in patients on chronic peritoneal dialysis. *Am J Nephrol.* (2008) 28:765–71. 10.1159/000128608 18434715

[19] Wen Y Guo Q Yang X Wu X Feng S Tan J High glucose concentrations in peritoneal dialysate are associated with all-cause and cardiovascular disease mortality in continuous ambulatory peritoneal dialysis patients. *Perit Dial Int.* (2015) 35:70–7. 10.3747/pdi.2013.00083 24293666 PMC4335930

[20] Radunz V Pecoits-Filho R Figueiredo AE Barretti P de Moraes TP. Impact of glucose exposure on outcomes of a nation-wide peritoneal dialysis cohort - results of the Brazpd Ii cohort. *Front Physiol.* (2019) 10:150. 10.3389/fphys.2019.00150 30890947 PMC6411763

[21] Abe M Hamano T Hoshino J Wada A Nakai S Hanafusa N Predictors of outcomes in patients on peritoneal dialysis: a 2-year nationwide cohort study. *Sci Rep.* (2019) 9:3967. 10.1038/s41598-019-40692-6 30850727 PMC6408436

[22] Park JY Cho JH Jang HM Kim YS Kang SW Yang CW Survival predictors in anuric patients on peritoneal dialysis: a prospective, multicenter, propensity score-matched cohort study. *PLoS One.* (2018) 13:e0196294. 10.1371/journal.pone.0196294 29694445 PMC5919016

[23] Hsieh C-Y Su C-C Shao S-C Sung S-F Lin S-J Yang Y-HK Taiwan’s national health insurance research database: past and future. *Clin Epidemiol.* (2019) 11:349. 10.2147/CLEP.S196293 31118821 PMC6509937

[24] Deyo RA Cherkin DC Ciol MA. Adapting a clinical comorbidity index for use with Icd-9-Cm administrative databases. *J Clin Epidemiol.* (1992) 45:613–9. 10.1016/0895-4356(92)90133-8 1607900

[25] Ma D Yan H Yang X Yu Z Ni Z Fang W. Abdominal aortic calcification score as a predictor of clinical outcome in peritoneal dialysis patients: a prospective cohort study. *BMC Nephrol.* (2020) 21:151. 10.1186/s12882-020-01822-9 32349690 PMC7191690

[26] Jiang N Zhang Z Fang W Qian J Mou S Ni Z. High peritoneal glucose exposure is associated with increased incidence of relapsing and recurrent bacterial peritonitis in patients undergoing peritoneal dialysis. *Blood Purif.* (2015) 40:72–8. 10.1159/000381663 26138314

[27] Williams JD Craig KJ Topley N Von Ruhland C Fallon M Newman GR Morphologic changes in the peritoneal membrane of patients with renal disease. *J Am Soc Nephrol.* (2002) 13:470–9. 10.1681/ASN.V132470 11805177

[28] Hara K Io H Wakabayashi K Maeda T Kanda R Nakata J Multicenter laparoscopic evaluation of the peritoneum in peritoneal dialysis patients. *Semin Dial.* (2020) 33:170–7. 10.1111/sdi.12870 32180272

[29] Aroeira LS Loureiro J Gonzalez-Mateo GT Fernandez-Millara V del Peso G Sanchez-Tomero JA Characterization of epithelial-to-mesenchymal transition of mesothelial cells in a mouse model of chronic peritoneal exposure to high glucose dialysate. *Perit Dial Int.* (2008) 28(Suppl 5):S29–33. 19008536

[30] Jiang J Chen P Chen J Yu X Xie D Mei C Accumulation of tissue advanced glycation end products correlated with glucose exposure dose and associated with cardiovascular morbidity in patients on peritoneal dialysis. *Atherosclerosis.* (2012) 224:187–94. 10.1016/j.atherosclerosis.2012.06.022 22857897

[31] Bartosova M Schaefer B Vondrak K Sallay P Taylan C Cerkauskiene R Peritoneal dialysis vintage and glucose exposure but not peritonitis episodes drive peritoneal membrane transformation during the first years of Pd. *Front Physiol.* (2019) 10:356. 10.3389/fphys.2019.00356 31001140 PMC6455046

[32] Zhang J Lu X Li H Wang S. Risk factors for mortality in patients undergoing peritoneal dialysis: a systematic review and meta-analysis. *Ren Fail.* (2021) 43:743–53. 10.1080/0886022X.2021.1918558 33913381 PMC8901278

[33] Lambie M Chess J Do JY Noh H Lee HB Kim YL Peritoneal dialysate glucose load and systemic glucose metabolism in non-diabetics: results from the global fluid cohort study. *PLoS One.* (2016) 11:e0155564. 10.1371/journal.pone.0155564 27249020 PMC4889040

