# Supplementary material for: Dialysis Duration and Glucose Exposure Amount Do Not Increase Mortality Risk in Peritoneal Dialysis Patients: A Population-Based Cohort Study From 2004 to 2012
Source: Front Med (Lausanne). 2022 Jun 28;9:897545. doi: 10.3389/fmed.2022.897545 (PMC9273817; doi:10.3389/fmed.2022.897545)
Supplement: Supplementary file 1 [file Data_Sheet_1.pdf]

Supplementary Table 1. Risk factors of all-cause mortality by different time windows.

Parameter	Two-year cohort (n=389)		Three-year cohort (n=495)		Four-year cohort (n=553)	
	HR (95% CI)	*p* value	HR (95% CI)	*p* value	HR (95% CI)	*p* value
Sex(female as reference)	0.78 (0.53−1.15)	0.21	0.84 (0.58−1.22)	0.36	0.95 (0.62−1.48)	0.83
Age(per year)	1.06 (1.04−1.07)	<0.05	1.05 (1.03−1.06)	<0.05	1.06 (1.04−1.08)	<0.05
Socioeconomic status						
Depended	Reference		Reference		Reference	
<20000 NTD	1.00 (0.53−1.83)	0.96	1.03 (0.55−1.90)	0.94	0.68 (0.32−1.45)	0.32
≥20000 NTD	0.99 (0.54−1.81)	0.99	1.03 (0.56−1.89)	0.94	1.07 (0.52−2.20)	0.85
Urbanization						
Rural	Reference		Reference		Reference	
Urban	0.97 (0.64−1.47)	0.90	0.95 (0.62−1.46)	0.82	1.15 (0.67−2.00)	0.61
Comorbidity						
Diabetes mellitus	1.39 (0.83−2.32)	0.21	1.42 (0.84−2.38)	0.19	1.96 (1.08−3.55)	0.03
Hypertension	0.82 (0.50−1.34)	0.43	0.73 (0.42−1.29)	0.21	0.98 (0.54−1.77)	0.95
Myocardial infarction	2.00 (0.70−5.74)	0.20	2.42 (0.86−6.85)	0.10	0.90 (0.12−6.65)	0.92
Congestive heart failure	1.18 (0.75−1.84)	0.49	1.19 (0.76−1.87)	0.42	1.23 (0.71−2.14)	0.46
Stroke	1.15 (0.60−2.19)	0.68	1.02 (0.52−1.97)	0.96	1.09 (0.48−2.48)	0.83
Gout	0.80 (0.49−1.31)	0.38	0.76 (0.46−1.26)	0.29	0.76 (0.43−1.33)	0.33
Peripheral vascular disease	1.54 (0.74−3.18)	0.25	1.41 (0.66−3.02)	0.37	3.32 (1.61−6.85)	<0.05
Charlson Comorbidity Index						
2	Reference		Reference		*	
3-4	0.23 (0.06−0.85)	0.03	0.34 (0.10−1.22)	0.10	Reference	
5-6	0.31(0.08−1.14)	0.08	0.63(0.18−2.20)	0.47	2.27 (1.08−4.80)	0.03
≥7	0.45 (0.11−1.80)	0.26	0.84 (0.22−3.29)	0.81	2.31 (0.90−5.91)	0.08
Time cohort						
2004-2005	Reference		Reference		Reference	
2006-2007	0.81 (0.52−1.25)	0.34	0.70 (0.44−1.10)	0.12	0.56 (0.32−0.99)	0.04
PD-related peritonitis (times)						
0	Reference		Reference		Reference	
1	1.06 (0.70−1.60)	0.78	1.19 (0.79−1.80)	0.41	1.17 (0.73−1.87)	0.52
≥2	1.50 (0.90−2.50)	0.12	1.69 (1.05−2.71)	0.03	0.95 (0.52−1.74)	0.86
PD duration (years)						
<1	Reference		Reference		Reference	

1-2 years	1.13 (0.78−1.60)	0.51	1.04 (0.67−1.61)	0.87	0.85 (0.48−1.51)	0.58
2-3 years	---		1.48 (0.96−2.28)	0.07	1.39 (0.80−2.43)	0.24
3-4 years	---		---		1.44 (0.79−2.62)	0.24
Mean PD glucose concentration (mg/dL)						
Low (<1.66)	Reference		Reference		Reference	
Moderate (1.66-1.96)	0.82 (0.53−1.27)	0.38	0.88 (0.58−1.35)	0.57	0.95 (0.58−1.55)	0.83
High (>1.96)	0.91 (0.58−1.43)	0.69	0.78 (0.49−1.23)	0.28	0.76 (0.44−1.31)	0.33

Abbreviation: HR, hazard ratio; CI, confidence interval; NTD, new Taiwan dollar; PD, peritoneal dialysis.

*No convergence for no death event in this subgroup.

Supplementary Table2-1. Death event number, observation period, and mortality concerning peritoneal dialysis exposure in the non-DM population by different time-window cohort.

Parameters	Patient number	Number of deaths	Observation period*, patient-years	Mortality rate (95% CI), per 1,000 patient-years	*p* value[&]
Two-year cohort	205	51	814.7	62.6 (48.9, 80.2)	
PD duration (year)					
<1	107	30	433.1	69.3 (54.7, 87.7)	Reference
1-2	98	21	381.5	55.0 (42.3, 71.7)	0.57
Mean PD glucose concentration[#] (mg/dL)					
Low (<1.63)	81	21	298.7	70.3 (55.6, 88.8)	Reference
Moderate (1.63-1.96)	62	17	242.9	70.0 (55.4, 88.5)	0.23
High (>1.96)	62	13	273.0	47.6 (35.8, 63.3)	0.11
Three-year cohort	283	54	947.7	57.0 (43.9, 73.9)	
PD duration (year)					
<1	96	20	332.4	60.1 (46.7, 77.5)	Reference
1-<2	84	12	294.4	40.8 (30.0, 55.4)	0.19
2-3	103	22	320.9	68.6 (54.1, 86.9)	0.35
Mean PD glucose concentration[#] (mg/dL)					
Low (<1.66)	107	21	331.5	63.3 (49.5, 81.0)	Reference
Moderate (1.66-1.96)	95	20	312.9	63.9 (50.0, 81.7)	0.70
High (>1.96)	81	13	303.3	42.9 (31.8, 57.8)	0.12
Four-year cohort	343	37	886.9	41.7 (30.8, 56.5)	
PD duration (year)					
<1.0	89	13	239.1	54.4 (41.7, 70.9)	Reference
1-<2	79	8	213.6	37.5 (27.2, 51.6)	0.22
2-<3	89	11	226.7	48.5 (36.6, 64.3)	0.76
3-4	86	5	207.5	24.1 (16.2, 35.9)	0.006
Mean PD glucose concentration[#] (mg/dL)					
Low (<1.65)	130	15	309.8	70.3 (55.6, 88.8)	Reference
Moderate (1.65-1.94)	114	13	291.8	70.0 (55.4, 88.5)	0.70
High (>1.94)	99	9	285.3	47.6 (35.8, 63.3)	0.12

Abbreviation: CI, confidence interval; DM, diabetes mellitus; PD, peritoneal dialysis.

*Calculated from the end of fixed time-windows, two-year, three-year, and four-year, respectively, to the time of death or 31 December, 2012.

[&]The different mortality rates by PD duration and glucose concentration in each time cohort were tested by Poisson regression model.

[#]Counted by using total accumulated glucose in PD dialysate divided by total accumulated PD solution volumes during PD treatment.

Supplementary Table 2-2. Death event number, observation period, and mortality concerning peritoneal dialysis exposure in the DM population by different time-window cohort.

Parameters	Patient number	Number of deaths	Observation period*, patient-years	Mortality rate (95% CI), per 1,000 patient-years	*p* value[&]
Two-year cohort	184	86	563.4	152.6 (130.3, 178.9)	
PD duration (year)					
<1	92	38	296.0	128.4 (108.0, 152.6)	Reference
1-2	92	48	267.4	179.5 (155.1, 207.8)	<0.001
Mean PD glucose concentration[#] (mg/dL)					
Low (<1.63)	47	24	135.2	177.6 (153.3, 205.7)	Reference
Moderate (1.63-1.96)	70	33	211.8	155.8 (133.1, 182.3)	<0.001
High (>1.96)	67	29	216.4	134.0 (113.1, 158.7)	<0.001
Three-year cohort	212	81	568.4	142.5 (120.9, 167.9)	
PD duration (year)					
<1	76	23	211.6	108.7 (90.1, 131.2)	Reference
1-<2	73	31	190.6	162.7 (139.5, 189.7)	<0.001
2-3	63	27	166.2	162.4 (139.3, 189.4)	<0.001
Mean PD glucose concentration[#] (mg/dL)					
Low (<1.66)	56	24	144.3	166.3 (142.8, 193.6)	Reference
Moderate (1.66-1.96)	73	31	183.0	169.4 (145.7, 196.9)	0.05
High (>1.96)	83	26	241.1	107.9 (89.3, 130.3)	<0.001
Four-year cohort	210	62	440.9	140.6 (119.2, 165.9)	
PD duration (year)					
<1.0	67	15	138.7	108.1 (89.5, 130.5)	Reference
1-<2	57	16	126.8	126.2 (106.0, 150.3)	<0.001
2-<3	51	15	110.5	135.7 (114.7, 160.6)	<0.001
3-4	35	16	64.8	246.8 (217.9, 279.6)	<0.001
Mean PD glucose concentration[#] (mg/dL)					
Low (<1.65)	52	19	104.1	182.5(157.8, 210.9)	Reference
Moderate (1.65-1.94)	74	23	139.6	164.8(141.4, 192.0)	<0.001
High (>1.94)	84	20	197.1	101.5(83.5, 123.2)	<0.001

Abbreviation: CI, confidence interval; DM, diabetes mellitus; PD, peritoneal dialysis.

*Calculated from the end of fixed time-windows, two-year, three-year, and four-year, respectively, to the time of death or 31 December, 2012.

[&]The different mortality rates by PD duration and glucose concentration in each time cohort were tested by Poisson regression model.

[#]Counted by using total accumulated glucose in PD dialysate divided by total accumulated PD solution volumes during PD treatment.

Supplementary Table3-1. Risk of mortality concerning peritoneal dialysis exposure in the non-DM population by different time-window cohort.

Parameters	Crude hazard ratio (95% CI)	Adjusted hazard ratio (95% CI)	*p*-value
Two-year cohort (n=205)			
PD duration (year)			0.85
<1	Reference	Reference	
1-2	0.80 (0.46, 1.39)	0.94 (0.50, 1.76)	0.85
Mean PD glucose concentration (mg/dL)			0.31
Low (<1.63)	Reference	Reference	
Moderate (1.63-1.96)	1.01 (0.53, 1.91)	0.61 (0.29, 1.28)	0.19
High (>1.96)	0.70 (0.30, 1.40)	0.61 (0.29, 1.31)	0.20
Three-year cohort (n=283)			
PD duration (year)			0.15
<1	Reference	Reference	
1-2	0.68 (0.33, 1.39)	0.58 (0.25, 1.31)	0.19
2-3	1.12 (0.61, 2.05)	1.29 (0.66, 2.51)	0.45
Mean PD glucose concentration (mg/dL)			0.38
Low (<1.66)	Reference	Reference	
Moderate (1.66-1.96)	1.03 (0.56, 1.9)	0.65 (0.32, 1.32)	0.23
High (>1.96)	0.7 (0.35, 1.4)	0.63 (0.29, 1.35)	0.23
Four-year cohort (n=343)			
PD duration(year)			0.10
<1	Reference	Reference	
1-2	0.69 (0.29, 1.66)	0.52 (0.19, 1.44)	0.21
2-3	0.88 (0.39, 1.96)	1.29 (0.52, 3.20)	0.59
3-4	0.44 (0.16, 1.23)	0.34 (0.11, 1.06)	0.06
Mean PD glucose concentration (mg/dL)			0.49
Low (<1.65)	Reference	Reference	
Moderate (1.65-1.94)	0.92 (0.44, 1.93)	0.72 (0.30, 1.72)	0.46
High (>1.94)	0.65 (0.28, 1.49)	0.58 (0.24, 1.43)	0.24

Abbreviation: CI, confidence interval; PD, peritoneal dialysis.

Model adjusted for age, sex, socioeconomic status, urbanization, comorbidity(hypertension, myocardial infarction, congestive heart failure, stroke, gout, and peripheral vascular disease), Charlson score, and PD-related peritonitis (categorized by none, once, and more than once).

Supplementary Table 3-2. Risk of mortality concerning peritoneal dialysis exposure in the DM population by different time-window cohort.

Parameters	Crude hazard ratio (95% CI)	Adjusted hazard ratio (95% CI)	*p*-value
Two-year cohort (n=184)			0.19
PD duration (year)			
<1	Reference	Reference	
1-2	1.41 (0.92, 2.16)	1.37 (0.86, 2.18)	0.19
Mean PD glucose concentration (mg/dL)			0.84
Low (<1.63)	Reference	Reference	
Moderate (1.63-1.96)	0.85 (0.5, 1.44)	0.92 (0.51, 1.65)	0.78
High (>1.96)	0.79 (0.46, 1.35)	1.09 (0.59, 2.02)	0.78
Three-year cohort (n=212)			
PD duration (year)			0.20
<1	Reference	Reference	
1-2	1.52 (0.89, 2.61)	1.48 (0.82, 2.66)	0.19
2-3	1.51 (0.86, 2.63)	1.74 (0.94, 3.22)	0.08
Mean PD glucose concentration (mg/dL)			0.68
Low (<1.66)	Reference	Reference	
Moderate (1.66-1.96)	1.01 (0.59, 1.72)	1.13 (0.64, 2.02)	0.67
High (>1.96)	0.68 (0.39, 1.19)	0.88 (0.47, 1.65)	0.69
Four-year cohort (n=210)			
PD duration(year)			0.005
<1	Reference	Reference	
1-2	1.21 (0.60, 2.45)	1.03 (0.46, 2.26)	0.95
2-3	1.27 (0.62, 2.59)	1.76 (0.80, 3.90)	0.16
3-4	2.27 (1.12, 4.59)	3.50 (1.58, 7.75)	0.002
Mean PD glucose concentration (mg/dL)			0.67
Low (<1.65)	Reference	Reference	
Moderate (1.65-1.94)	0.89 (0.48, 1.63)	1.14 (0.59, 2.21)	0.70
High (>1.94)	0.59 (0.31, 1.10)	0.83 (0.40, 1.72)	0.63

Abbreviation: CI, confidence interval; PD, peritoneal dialysis.

Model adjusted for age, sex, socioeconomic status, urbanization, comorbidity(hypertension, myocardial infarction, congestive heart failure, stroke, gout,and peripheral vascular disease), Charlson score, and PD-related peritonitis (categorized by none, once, and more than once).

Supplementary Table 4. Risk of mortality concerning peritoneal dialysis exposure by different time-window cohorts by cause-specific Cox regression model#

Parameters	Crude hazard ratio (95% CI)	Adjusted hazard ratio (95% CI)	*p*-value
Two-year cohort (n=389)			
PD duration (year)			0.94
<1	Reference	Reference	
1-2	1.14 (0.82, 1.60)	0.98 (0.66, 1.46)	0.94
Mean PD glucose concentration (mg/dL)			0.69
Low (<1.63)	Reference	Reference	
Moderate (1.63-1.96)	1.05 (0.70, 1.57)	0.84 (0.52, 1.35)	0.47
High (>1.96)	0.86 (0.57, 1.31)	0.83 (0.51, 1.35)	0.44
Three-year cohort (n=495)			
PD duration (year)			0.54
<1	Reference	Reference	
1-2	1.13 (0.74, 1.73)	0.95 (0.59, 1.51)	0.82
2-3	1.26 (0.84, 1.90)	1.22 (0.77, 1.92)	0.40
Mean PD glucose concentration (mg/dL)			0.69
Low (<1.66)	Reference	Reference	
Moderate (1.66-1.96)	1.10 (0.74, 1.65)	0.84 (0.54, 1.33)	0.46
High (>1.96)	0.80 (0.52, 1.22)	0.83 (0.52, 1.34)	0.45
Four-year cohort (n=553)			
PD duration(year)			0.67
<1	Reference	Reference	
1-2	0.96 (0.56, 1.66)	0.76 (0.41, 1.41)	0.38
2-3	1.03 (0.60, 1.76)	1.13 (0.63, 2.03)	0.69
3-4	1.03 (0.58, 1.81)	0.96 (0.49, 1.88)	0.91
Mean PD glucose concentration (mg/dL)			0.65
Low (<1.65)	Reference	Reference	
Moderate (1.65-1.94)	1.02 (0.64, 1.63)	0.94 (0.55, 1.61)	0.83
High (>1.94)	0.76 (0.46, 1.25)	0.77 (0.44, 1.36)	0.37

Renal transplant as a competing event

Abbreviation: CI, confidence interval; PD, peritoneal dialysis.

Model adjusted for age, sex,socioeconomic status, urbanization, comorbidity (diabetes mellitus, hypertension, myocardial infarction, congestive heart failure, stroke, gout, and peripheral vascular disease), Charlson score, and PD-related peritonitis(categorized by none, once, and more than once), vascular access type (categorized by fistula, graft, cuffed permcatheter, and temporary double-lumen catheter), and HD center size (categorized by tertiary hospital, region hospital, district hospital, and clinic)
